# Personalized computational modeling of left atrial geometry and transmural myofiber architecture

**DOI:** 10.1016/j.media.2018.04.001

**Published:** 2018-07

**Authors:** Thomas E. Fastl, Catalina Tobon-Gomez, Andrew Crozier, John Whitaker, Ronak Rajani, Karen P. McCarthy, Damian Sanchez-Quintana, Siew Y. Ho, Mark D. O’Neill, Gernot Plank, Martin J. Bishop, Steven A. Niederer

**Affiliations:** a Department of Biomedical Engineering, King’s College London, London, United Kingdom; b Institute of Biophysics, Medical University of Graz, Graz, Austria; c Department of Cardiology, Guy’s and St Thomas’ Hospitals, London, United Kingdom; d Cardiac Morphology Unit, Royal Brompton Hospital, London, United Kingdom; e Department of Anatomy and Cell Biology, University of Extremadura, Badajoz, Spain

**Keywords:** Personalized computational modeling, Finite element method, Atrial fiber architecture, Atrial electrophysiology

## Abstract

Atrial fibrillation (AF) is a supraventricular tachyarrhythmia characterized by complete absence of coordinated atrial contraction and is associated with an increased morbidity and mortality. Personalized computational modeling provides a novel framework for integrating and interpreting the role of atrial electrophysiology (EP) including the underlying anatomy and microstructure in the development and sustenance of AF. Coronary computed tomography angiography data were segmented using a statistics-based approach and the smoothed voxel representations were discretized into high-resolution tetrahedral finite element (FE) meshes. To estimate the complex left atrial myofiber architecture, individual fiber fields were generated according to morphological data on the endo- and epicardial surfaces based on local solutions of Laplace’s equation and transmurally interpolated to tetrahedral elements. The influence of variable transmural microstructures was quantified through EP simulations on 3 patients using 5 different fiber interpolation functions. Personalized geometrical models included the heterogeneous thickness distribution of the left atrial myocardium and subsequent discretization led to high-fidelity tetrahedral FE meshes. The novel algorithm for automated incorporation of the left atrial fiber architecture provided a realistic estimate of the atrial microstructure and was able to qualitatively capture all important fiber bundles. Consistent maximum local activation times were predicted in EP simulations using individual transmural fiber interpolation functions for each patient suggesting a negligible effect of the transmural myofiber architecture on EP. The established modeling pipeline provides a robust framework for the rapid development of personalized model cohorts accounting for detailed anatomy and microstructure and facilitates simulations of atrial EP.

## 1 Introduction

Atrial fibrillation (AF) is a supraventricular tachyarrhythmia characterized by uncoordinated atrial activation with consequent deterioration of mechanical function. Affecting an estimated 33 million people worldwide (Rahman et al., 2014), AF is the most common arrhythmia and is associated with an increased long-term risk of other cardiovascular diseases. Personalized computational modeling provides a novel framework for integrating and interpreting the role of electrophysiology (EP) in the development and progression of AF (Trayanova, 2014, Trayanova, Chang, 2016). Numerous atrial models of different species, varying complexity and diverse applications have been generated over the last decades and used to investigate medical questions, however, a processing pipeline for standardized generation of personalized atrial computer models remains unavailable (Seemann, Höper, Sachse, Dössel, Holden, Zhang, 2006, Jernigan, Buckner, Eischen, Cormier, 2007, Zhang, Gay, 2008, Hunter, Liu, Lu, Wang, Schilling, 2012, Krueger, Seemann, Rhode, Keller, Schilling, Arujuna, Gill, O’Neill, Razavi, Dössel, 2013, Gonzales, Sturgeon, Krishnamurthy, Hake, Jonas, Stark, Rappel, Narayan, Zhang, Segars, McCulloch, 2013, Satriano, Bellini, Vigmond, Di Martino, 2013, Adeniran, MacIver, Garratt, Ye, Hancox, Zhang, 2015, Ferrer, Sebastián, Sánchez-Quintana, Rodríguez, Godoy, Martínez, Saiz, 2015, Moyer, Norton, Ferguson, Holmes, 2015, McDowell, Zahid, Vadakkumpadan, Blauer, MacLeod, Trayanova, 2015, Zhao, Hansen, Csepe, Lim, Wang, Williams, Mohler, Janssen, Weiss, Hummel, Fedorov, 2015, Zhao, Hansen, Wang, Csepe, Sul, Tang, Yuan, Li, Bratasz, Powell, Kilic, Mohler, Janssen, Weiss, Simonetti, Hummel, Fedorov, 2017, Varela, Colman, Hancox, Aslanidi, 2016). While an increased image resolution facilitates the generation of detailed anatomical models of the atria (Bishop, Rajani, Plank, Gaddum, Carr-White, Wright, O’Neill, Niederer, 2016, Pashakhanloo, Herzka, Ashikaga, Mori, Gai, Bluemke, Trayanova, McVeigh, 2016, Zhao, Hansen, Csepe, Lim, Wang, Williams, Mohler, Janssen, Weiss, Hummel, Fedorov, 2015, Zhao, Hansen, Wang, Csepe, Sul, Tang, Yuan, Li, Bratasz, Powell, Kilic, Mohler, Janssen, Weiss, Simonetti, Hummel, Fedorov, 2017), standardized assignment of the complex atrial myofiber architecture remains challenging. Computational studies in the atria have demonstrated the influence of structural anisotropy on EP substantiating the importance of the atrial fiber orientation in finite element (FE) simulations (Krueger, Schmidt, Tobón, Weber, Lorenz, Keller, Barschdorf, Burdumy, Neher, Plank, Rhode, Seemann, Sanchez-Quintana, Saiz, Razavi, Dössel, 2011, Krueger, Seemann, Rhode, Keller, Schilling, Arujuna, Gill, O’Neill, Razavi, Dössel, 2013, Ferrer, Sebastián, Sánchez-Quintana, Rodríguez, Godoy, Martínez, Saiz, 2015).

Significant limitations of atrial *in vivo* imaging, due to its thin-walled phenotype, have motivated comprehensive anatomical and morphological *ex vivo* studies aiming to characterize the atrial fiber architecture qualitatively over the endo- and epicardial surfaces (Ho, Sanchez-Quintana, Cabrera, Anderson, 1999, Ho, Anderson, Sánchez-Quintana, 2002, Cabrera, Ho, Climent, Sánchez-Quintana, 2008, Ho, Sánchez-Quintana, 2009). Additional *ex vivo* quantifications of the atrial fiber orientation include serial surface macroscopy (Zhao, Butters, Zhang, Pullan, LeGrice, Sands, Smaill, 2012, Zhao, Butters, Zhang, LeGrice, Sands, Smaill, 2013), micro-computed tomography (CT) (Varela, Zhao, Aslanidi, 2013, Zhao, Hansen, Csepe, Lim, Wang, Williams, Mohler, Janssen, Weiss, Hummel, Fedorov, 2015, Stephenson, Atkinson, Kottas, Perde, Jafarzadeh, Bateman, Iaizzo, Zhao, Zhang, Anderson, Jarvis, Dobrzynski, 2017), diffusion tensor magnetic resonance imaging (MRI) (Pashakhanloo et al., 2016) and contrast-enhanced MRI (Zhao et al., 2017).

The majority of atrial modeling studies have incorporated the structural anisotropy using rule-based approaches to qualitatively represent observed fiber morphologies in the literature. Due to the complex structure of the atrial fiber architecture, however, applied rules have been manually attributed to specific atrial regions and structures (Harrild, Henriquez, 2000, Vigmond, Ruckdeschel, Trayanova, 2001, Jacquemet, Virag, Ihara, Dang, Blanc, Zozor, Vesin, Kappenberger, Henriquez, 2003, Seemann, Höper, Sachse, Dössel, Holden, Zhang, 2006, Tobón, Ruiz-Villa, Heidenreich, Romero, Hornero, Saiz, 2013, Gonzales, Sturgeon, Krishnamurthy, Hake, Jonas, Stark, Rappel, Narayan, Zhang, Segars, McCulloch, 2013, Ferrer, Sebastián, Sánchez-Quintana, Rodríguez, Godoy, Martínez, Saiz, 2015, Moyer, Norton, Ferguson, Holmes, 2015). Although the methods presented in the individual studies have appropriately reflected the underlying atrial fiber distribution, these approaches are not suitable for large patient cohorts. To generate personalized computational models of the atria including the fiber orientation for multiple patient geometries, a variety of rule-based semi-automatic approaches have been proposed (Werner, Sachse, Dössel, 2000, Hermosillo, 2008, Krueger, Schmidt, Tobón, Weber, Lorenz, Keller, Barschdorf, Burdumy, Neher, Plank, Rhode, Seemann, Sanchez-Quintana, Saiz, Razavi, Dössel, 2011, Labarthe, Coudiere, Henry, Cochet, 2012, Satriano, Bellini, Vigmond, Di Martino, 2013). Furthermore, atlas-based methods, in which a number of distinct landmarks are used to warp an existing atrial fiber orientation defined on a source geometry onto a particular target geometry, are becoming increasingly popular (McDowell, Vadakkumpadan, Blake, Blauer, Plank, MacLeod, Trayanova, 2012, McDowell, Vadakkumpadan, Blake, Blauer, Plank, MacLeod, Trayanova, 2013, Satriano, Bellini, Vigmond, Di Martino, 2013, McDowell, Zahid, Vadakkumpadan, Blauer, MacLeod, Trayanova, 2015). Both techniques estimate the atrial fiber architecture, however, manual intervention will increase intra- and interobserver variability as well as limit reproducibility. At present there are no clinically applicable automated methods to either generate anatomically detailed personalized atrial computer geometries or reliably estimate the atrial fiber architecture *in vivo*.

An integrative pipeline to generate personalized computational models of the left atrium (LA) suitable for FE simulations of atrial EP is presented in this article. Statistics-based image segmentation of high-resolution coronary CT angiography (CTA) data was performed and the smoothed voxel representations subsequently discretized into high-resolution and high-fidelity tetrahedral FE meshes. Morphological data informed the qualitative estimation of the complex left atrial fiber architecture, primarily based on local solutions of Laplace’s equation. The automated modeling pipeline was exercised on 3 patient cases, while electrophysiological FE simulations were performed to analyze the influence of the transmural tissue microstructure on the local activation time (LAT).

## 2 Methods

**Fig. 1:**
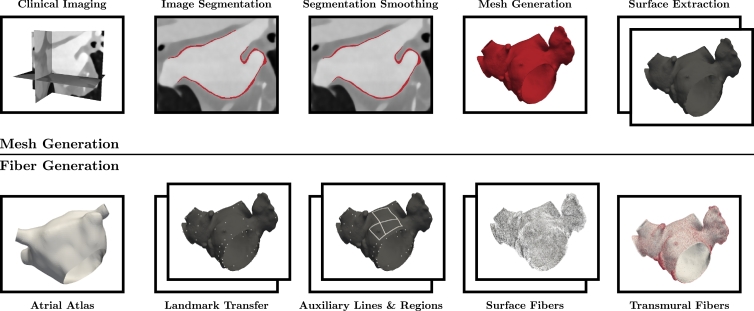
Personalized computational modeling pipeline for left atrial electrophysiology separated into the mesh generation phase (top) and the subsequently performed fiber generation phase (bottom). Individual boxes illustrate representative generation processes, while two boxes indicate processes on multiple surfaces, i.e., endo- and epicardium.

The automated modeling pipeline for generating detailed personalized computational models of the LA is illustrated in Fig. 1. Preliminary results have been published in Fastl et al. (2016). All details for the proposed algorithm are provided in this paper. Retrospective analysis of 3 patient cases (see Table 1 for baseline demographics) who underwent a clinically indicated coronary CTA to exclude coronary artery disease was performed to exercise the proposed atrial modeling pipeline. Selected personalized computational models of the LA including the estimated fiber architectures are available online (http://doi.org/doi:10.18742/RDM01-289).

**Table 1:** Baseline demographics for retrospectively analyzed patient cohort. Abbreviations: M (male), F (female), HLD (hyperlipidemia), PAF (paroxysmal atrial fibrillation) and SSS (sick sinus syndrome).

	Sex	Age [yr]	Comorbidities
Patient I	M	35	HLD
Patient II	F	48	NIL
Patient III	F	54	PAF, SSS

### 2.1 Clinical imaging

The applied coronary CTA protocol has been described previously in Bishop et al. (2016). Briefly, coronary CTA data were acquired at Guy’s and St Thomas’ NHS Foundation Trust using a Philips 256 iCT scanner (Philips Healthcare, Amsterdam, Netherlands). The acquired coronary CTA data were reconstructed at 75% of the RR interval (most likely corresponding to the left atrial conduit phase) using an iterative reconstruction (iDose level 4) with 0.80 mm slice thickness, 0.40 mm slice increment, 250.00 mm field of view (approximate size), 512 × 512 matrix and a smooth reconstruction kernel (Donal et al., 2016).

### 2.2 Image segmentation

**Fig. A1:**
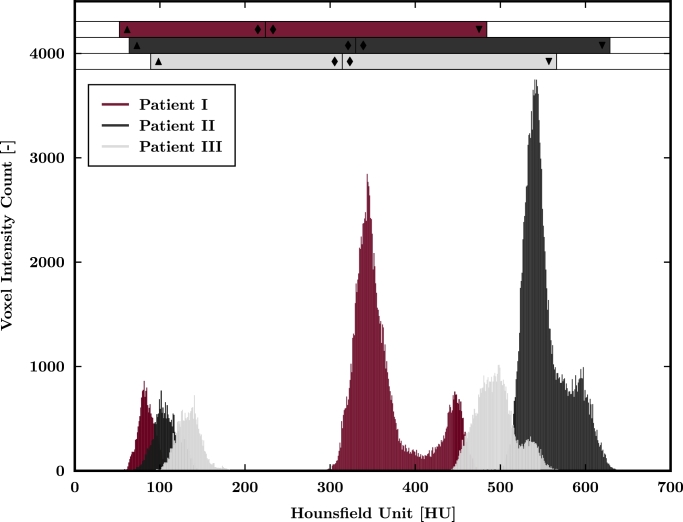
Voxel intensity histograms of the combined 6 sample regions (3 sample regions in each, the atrial blood pool and the left ventricular myocardium) for all patients. The superimposed bars diagrams indicate the calculated personalized intensity thresholds of the left atrial myocardium (▲ - ♦) and the left atrial cavity (♦ - ▼).

Based on the assumption of comparable radiodensities in the atrial and ventricular myocardium (Bishop et al., 2016), a regionally tagged segmentation of the LA was generated using the software package Seg3D (Center for Integrative Biomedical Computing, 2016). Within the image space 3 sample regions of approximately equal size were selected in both the left ventricular myocardium and the left atrial cavity, for which combined mean values and standard deviations were calculated (see voxel intensity histograms including calculated thresholds in Fig. A1). Coronary CTA images were processed using a 4-point median filter to enhance the image contrast and manually cropped to encapsulate the LA and reduce unnecessary computational overhead. The left atrial myocardium and the left atrial blood pool were thresholded utilizing their corresponding statistical sample mean value reduced or increased by 3 times the sample standard deviation, respectively. The continuous transition between the left atrial myocardium and the left atrial blood pool required a common threshold value chosen as the average of both statistical mean values of the combined sample regions. Furthermore, the viable left atrial myocardium was restricted to a maximum thickness of 3 mm to separate any neighboring structures, e.g., right and left ventricles, right atrium, aorta and coronary sinus, with similar Hounsfield units (Dössel, Krueger, Weber, Wilhelms, Seemann, 2012, Whitaker, Rajani, Chubb, Gabrawi, Varela, Wright, Niederer, O’Neill, 2016).

Initial dilation of the segmented left atrial blood pool by 1 voxel was performed to obtain a minimum wall thickness. The subsequent iterative dilations combined with logical operations ( ∩ ) on the viable atrial myocardium provided the raw segmentation of the left atrial wall. Some minor cosmetic operations, e.g., smoothing and filling operations, were applied to obtain the final segmentation of the left atrial myocardium Ω_MC_. Standardized mitral valve (MV) exclusion was performed utilizing a binary spherical shell Ω_SS_ generated by manual specification of 3 points around the MV annulus and removal of voxels distal from the LA. Pulmonary veins (PVs) were cropped using multiple binary cylindrical disks $\Omega_{\text{CD}}=\sum_{i=1}^{n}\Omega_{\text{CD}}^{i},$ with i=1,⋯,n, where *n* is the number of PVs present, by manual identification of 3 points on each vein *i* and removal of voxels distal from the LA. Both geometries, the spherical shell as well as the cylindrical disks, were subsequently employed to generate tags for the MV annulus, i.e., $\Omega_{\text{MV}}=\Omega_{\text{MC}}\cap \Omega_{\text{SS}},$ and the ends of the PVs, i.e., $\Omega_{\text{PV}}=\Omega_{\text{MC}}\cap \Omega_{\text{CD}},$ while the representation of the left atrial wall becomes $\Omega_{\text{AW}}=\left[\Omega_{\text{MC}}⊕\left(\Omega_{\text{MV}}\cup \Omega_{\text{PV}}\right)\right],$ with ⊕ denoting the XOR operation.

### 2.3 Segmentation smoothing

A combined smoothing and upsampling algorithm was applied to the anatomically tagged voxel representation attenuating staircase effects and providing an isotropic segmentation with sufficient spatial resolution for computational FE mesh generation (Crozier et al., 2016). While a variational method was employed to correct for low resolution artifacts of the surface, smoothing was achieved by minimization of a high-order penalty restricting the maximum displacement of the reference surface to 50% of the voxel size ensuring that the resulting isosurface is within the error margin of the imaging modality. The resulting surface encompassing the left atrial geometry was resampled providing an image segmentation with a consistently isotropic 100 µm voxel resolution.

### 2.4 Mesh generation

A high-fidelity and high-resolution mesh of the LA was created using the Octree-based mesh generation software Tarantula (CAE Software Solutions, Eggenburg, Austria), which builds unstructured and locally refined tetrahedral FE meshes and maps classification tags from the input segmentation onto the generated mesh. Identification of the endo- and epicardium including the corresponding surface meshes of the LA relied on a breadth-first search algorithm implemented in MATLAB (The MathWorks, Inc., Natick, United States).

### 2.5 Image registration

A major advantage of the proposed method to generate detailed personalized computational models of the LA including the complex fiber architecture is the automated transfer of predefined landmarks from an average atrial geometry to the personalized atrial geometry. This average atrial geometry was generated by combination of 30 MRI datasets and manual segmentation of the endocardium (Tobon-Gomez et al., 2015).

Endo- and epicardial surface meshes were decimated to approximately 10% of the reference element number using the Computational Geometry Algorithms Library facilitating a tractable image registration (Cacciola, 2017). The resulting surface meshes were manually initialized to match the average atrial geometry and subsequently registered using the software Deformetrica (Durrleman et al., 2014). A nearest neighbor approach was applied to transfer 122 predefined landmarks on the average atrial geometry to the corresponding decimated and registered surface mesh (the maximum potential mapping error, the average distance between nodes of the atrial atlas and the nearest nodes on the registered surface meshes of all patients, was evaluated as 0.52 mm, approximately equal to the image resolution). During the image registration process the mesh topology was maintained, hence a final nearest neighbor transfer of the landmarks from the decimated to the personalized atrial surface meshes provided their location. An additional 62 landmarks were calculated around the PVs, MV, left atrial appendage (LAA) and interatrial septum using the spatial information of already transferred landmarks. Minor manual adjustment of individual landmarks around the PVs, MV and LAA was performed where necessary as a consequence of the large structural variability.

### 2.6 Surface fiber generation

**Fig. 2:**
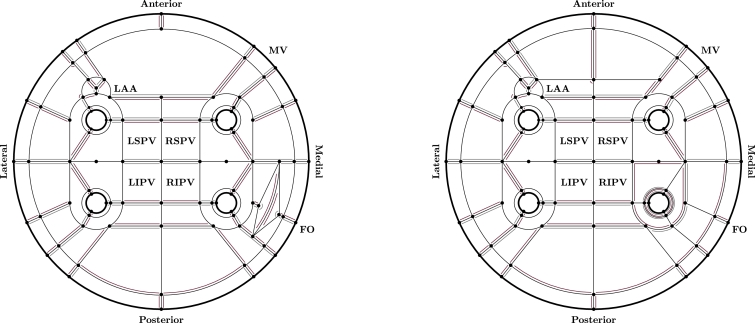
Idealized unfolded representation of the endo- (left panel) and epicardial (right panel) surfaces of the left atrium indicating individual landmarks (only constituting landmarks of auxiliary lines are provided), auxiliary lines and atrial regions. Boundary conditions to generate regional surface fibers are provided in red and gray corresponding to (2.1) and (2.2), respectively, while (2.3) was applied to any remaining boundary. Abbreviations: LAA (left atrial appendage), MV (mitral valve), FO (fossa ovalis), RSPV (right superior pulmonary vein), RIPV (right inferior pulmonary vein), LSPV (left superior pulmonary vein) and LIPV (left inferior pulmonary vein). (For interpretation of the references to colour in this figure legend, the reader is referred to the web version of this article.)

Landmarks on the left atrial surfaces constituted a predefined network of 272 auxiliary lines, which subdivided both endo- and epicardial surfaces into 151 atrial regions (see Fig. 2) similar to the approaches in Hunter et al. (2012) and Teh et al. (2012). The auxiliary lines were calculated using an implementation of the Dijkstra algorithm applied on preselected corridors utilizing the geodesic paths between the corresponding landmarks. Then, a region growing algorithm identified the atrial regions Ω, on which Laplace’s equation

(1) $$\nabla^{2}u=0$$

was solved within the Cardiac Arrhythmia Research Package (Vigmond et al., 2003) using boundary conditions applied to *∂Ω*_I_, *∂Ω*_II_ and *∂Ω*_R_ representing the bordering auxiliary lines (see Fig. 2), i.e.,

(2) $${u|}_{\partial \Omega_{I}}{=0,\;u|}_{\partial \Omega_{\text{II}}}=1,\;\frac{\partial u}{\partial n}{|}_{\partial \Omega_{R}}=0.$$

The normalized gradient of the obtained scalar field ∇*u*/‖∇*u*‖ evaluated at the surface triangle centers represented the modeled fiber orientation and estimated the morphologically observed atrial fiber architecture. The calculated fiber vectors were complemented with the fiber vectors of adjacent elements where necessary to obtain a full representation of the personalized fiber field on the endo- and epicardial surfaces of the LA. Interregional fiber smoothing using an orientation-based neighborhood approach was applied to triangular elements in the proximity of the auxiliary lines. Finally, out-of-plane fibers due to the smoothing procedure were projected onto the tangential plane of the atrial surface followed by vector normalization.

### 2.7 Transmural fiber generation

**Fig. 3:**
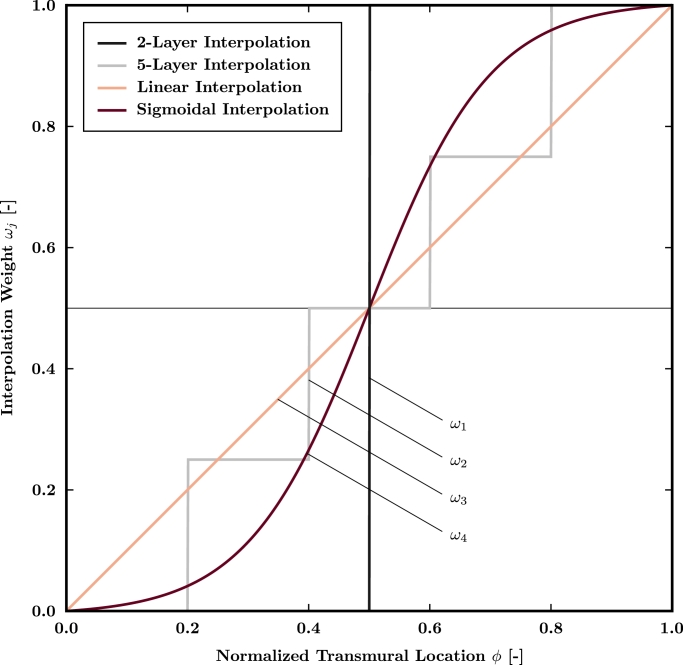
Employed fiber interpolation functions providing an interpolation weight *ω_j_* for each tetrahedral element dependent on the normalized transmural element location *ϕ* evaluated at the tetrahedral element center.

Fiber direction vectors for tetrahedral elements were obtained by transmural interpolation (see Fig. 3) of the estimated surface fiber field according to a correspondence map. Therefore, (1) was solved on the tetrahedral FE mesh Ω with boundary conditions in (2) applied to *∂Ω*_I_, *∂Ω*_II_ and *∂Ω*_R_ representing the endocardial, epicardial and remaining boundary surfaces, respectively. Starting from the tetrahedral element center, endo- and epicardial surface triangles were identified by tracking the unit field lines evaluated as ∇*u*/‖∇*u*‖. An element average of the nodal solutions *u* representing the normalized transmural location *ϕ* was calculated and utilized to evaluate an interpolation weight *ω_j_*, where *j* represents a mathematical function as defined below.

The influence of variable transmural microstructures on LAT was quantified using 4 different transmural interpolation functions *ω_j_* graphically shown in Fig. 3. Since anatomical and morphological studies have indicated a multi-layer structure within the left atrial myocardium, though the exact number of layers remains unknown, 2-layer (3), 5-layer (4), linear (5) and sigmoidal (6) transmural fiber interpolation functions were chosen given as

(3) $$\begin{array}{cc} \omega_{1}\left(\phi \right) & =\left\{ \begin{array}{cc} 0.00 & \;\text{if}\;\phi \leq 0.50\\ 1.00 & \;\text{if}\;\phi >0.50 \end{array}\right., \end{array}$$

(4) $$\begin{array}{cc} \omega_{2}\left(\phi \right) & =\left\{ \begin{array}{cc} 0.00 & \;\text{if}\;\phi \leq 0.20\\ 0.25 & \;\text{if}\;0.20<\phi \leq 0.40\\ 0.50 & \;\text{if}\;0.40<\phi \leq 0.60\\ 0.75 & \;\text{if}\;0.60<\phi \leq 0.80\\ 1.00 & \;\text{if}\;\phi >0.80 \end{array}\right., \end{array}$$

(5) $$\begin{array}{cc} \omega_{3}\left(\phi \right) & =\phi , \end{array}$$

(6) $$\begin{array}{cc} \omega_{4}\left(\phi \right) & =\frac{1}{2}\tanh\left(5\left(\phi -0.5\right)\right), \end{array}$$

respectively, where tanh  represents the hyperbolic tangent. The fiber direction in all tetrahedral elements was assigned based on the estimated direction vectors of the corresponding surface triangles according to

(7) $$f^{i}=\frac{\left(1-\omega_{j}^{i}\right)f_{\text{ED}}^{i}+\omega_{j}^{i}f_{\text{EP}}^{i}}{∥\left(1-\omega_{j}^{i}\right)f_{\text{ED}}^{i}+\omega_{j}^{i}f_{\text{EP}}^{i}∥},$$

where $f_{\text{ED}}^{i}$ and $f_{\text{EP}}^{i}$ denote the surface fiber direction vectors on the endo- and epicardium for element *i*, respectively. Finally, all tetrahedral element fiber direction vectors were projected onto a plane orthogonal to the associated unit field line.

### 2.8 Electrophysiology simulations

In all patients cellular EP was described using the Courtemanche–Ramirez–Nattel (CRN) model to simulate the human atrial action potential (Courtemanche et al., 1998). The intracellular current flow responsible for the spread of electrical activation in the atrial myocardium was calculated using the monodomain equation

(8) $$\beta_{m}C_{m}\frac{dV_{m}}{dt}+\beta_{m}I_{\text{ion}}\left(V_{m},\eta \right)=\nabla \cdot \left(\sigma_{m}\nabla V_{m}\right)+I_{\text{tr}},$$

where *β*_m_ is the membrane surface-to-volume ratio, *C*_m_ is the membrane capacity, *V*_m_ is the transmembrane potential, *I*_ion_ is the density of the total ionic currents as a function of *V*_m_ and a set of state variables ***η***, *I*_tr_ is a transmembrane stimulus current and ***σ***_m_ represents the monodomain conductivity tensor. Single cell stimulation on the standard CRN model using 1000 beats with a basic cycle length of 1000 ms was performed prior to EP simulations to obtain steady state conditions. The conduction velocities in the LA were chosen as 1.20 and 0.40  m/s in longitudinal and transversal direction, respectively, leading to an anisotropy ratio of 3/1, well within the range of reported values for healthy patients (Dimitri, Ng, Brooks, Kuklik, Stiles, Lau, Antic, Thornton, Saint, McEvoy, Antic, Kalman, Sanders, 2012, Zemlin, Herzel, Ho, Panfilov, 2001). To match the reported conduction velocities in the EP simulations, the monodomain conductivity tensor ***σ***_m_ was iteratively fitted using the method described in Mendonca Costa et al. (2013). The Cardiac Arrhythmia Research Package was employed to numerically solve (8) via the FE method using a global time step of 10 µs (Vigmond et al., 2008). Regionally confined epicardial stimulation around the Bachmann’s bundle (BB) was applied to mimic the physiological signal transduction from the right atrium (Markides et al., 2003). The LAT was recorded once the transmembrane potential *V*_m_ exceeded a threshold of −20mV. Besides anisotropic EP simulations utilizing the 4 different transmural microstructures introduced, an isotropic reference EP simulation was performed for each patient. To model the non-physiological isotropic electrical material, the anisotropic monodomain conductivity tensor ***σ***_m_ was combined with a random fiber architecture.

## 3 Results

The automated personalized modeling pipeline was exercised on 3 patient cases (see Table 1) to demonstrate its ability to generate detailed FE models including a qualitative representation of the left atrial fiber architecture. The influence of a variable transmural microstructure utilizing different transmural fiber interpolation functions on the LAT was quantified using electrophysiological FE simulations.

### 3.1 Computational model generation

Acquired coronary CTA images (see Table 2 for voxel dimensions) were retrospectively analyzed and voxel intensity histograms of the combined 6 individual sample regions selected within the left atrial blood pool and the left ventricular myocardium for individual patients are presented in Fig. A1. Based on the obtained histograms, personalized intensity thresholds for the left atrial myocardium and the left atrial cavity were calculated and are specified in Table 2. Furthermore, important characteristics on the generated high-fidelity and high-resolution FE meshes are presented in Table 2. While consistent average element edge lengths of approximately 120 µm were achieved in all patients due to isotropic segmentation resampling, a large variation in myocardial tissue volume (assumed as the volume of the FE mesh) between patients was observed. This explained the large differences in node and element numbers between individual patients.

**Table 2:** Clinical imaging, image segmentation and mesh generation characteristics for retrospectively analyzed patient models. The voxel dimensions VD (prior to segmentation smoothing and upsampling) depend on the chosen imaging parameters. The segmentation intensity thresholds LT, MT and UT represent the lower, mid and upper thresholds to identify the left atrial myocardium and the left atrial cavity, respectively. Finite element mesh properties including the number of nodes #_nodes_, number of elements #_elements_, mesh volume VL and average element edge length EL are provided.

	VD [mm]	LT [HU]	MT [HU]	UT [HU]	#_nodes_ [–]	#_elements_ [–]	VL [cm^3^]	EL [µm]
Patient I	0.35 × 0.35 × 0.40	52.26	223.97	484.26	21, 868, 400	121, 207, 799	18.05	120.72
Patient II	0.25 × 0.25 × 0.40	63.74	330.01	628.88	14, 352, 781	79, 067, 310	11.79	120.84
Patient III	0.39 × 0.39 × 0.40	89.04	314.27	566.27	8, 736, 713	46, 154, 317	6.43	116.85

### 3.2 Estimated fiber architecture

The novel algorithm to estimate the left atrial fiber architecture qualitatively captured the complex arrangement of fiber bundles in the LA. Although interpatient variation has been observed in morphological images, the predominant features of the left atrial fiber architecture are preserved.

**Fig. 4:**
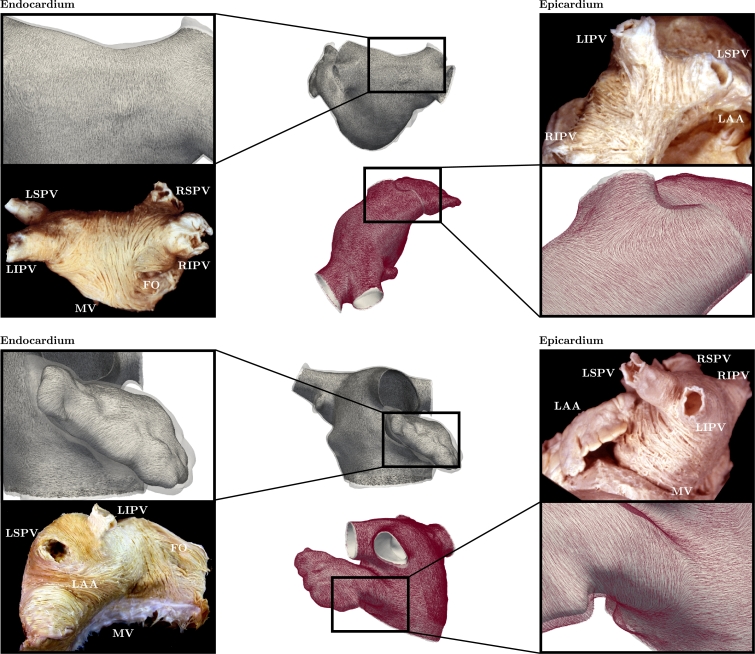
Comparison between modeled left atrial fiber orientation in Patient III and morphological images on the endocardium (left panel), shown in everted and horizontally flipped state (cf., e.g., Ho et al. (1999) and Cabrera et al. (2008)), and the epicardium (right panel). Specifically, comparisons on the endocardial posterior region (top left), the endocardial left atrial appendage (bottom left), the epicardial posterior region including the pulmonary veins (top right) and the epicardial lateral region (bottom right) are shown. Abbreviations: LAA (left atrial appendage), MV (mitral valve), FO (fossa ovalis), RSPV (right superior pulmonary vein), RIPV (right inferior pulmonary vein), LSPV (left superior pulmonary vein) and LIPV (left inferior pulmonary vein).

The atrial fiber structure on the anterior endocardium was dominated by the septoatrial bundle ascending oblique from the anterior interatrial raphe. The septoatrial bundle split up into 3 major fascicles to encircle the LAA and combine with longitudinal fibers at the posterior portion of the LA between the PVs (see top left comparison in Fig. 4). Endocardial fibers at the PV orifices transfered into a circular fiber arrangement around all PVs, while circular fibers were also observed around the MV and the LAA (see bottom left comparison in Fig. 4). An elliptical fiber orientation was obtained at the left atrial portion of the interatrial septum representing the fossa ovalis (FO).

The atrial fiber structure on the anterior epicardium was dominated by the septopulmonary bundle (see top right comparison in Fig. 4) and the BB. While the fascicles of the BB, which ran parallel to the atrioventricular groove, encircled the LAA, oblique and longitudinal fibers of the septopulmonary bundle proceeded longitudinally at the posterior wall of the LA besides fanning out to entwine the PVs. Similar to the endocardium, circular fiber arrangements were observed around the PVs, with the exception of longitudinal fibers at the right inferior PV (RIPV) (see top right comparison in Fig. 4), MV and LAA (see bottom right comparison in Fig. 4) on the epicardium. The complex epicardial region around the FO was simplified, hence a longitudinal fiber orientation was obtained.

### 3.3 Electrophysiology simulations

**Fig. 5:**
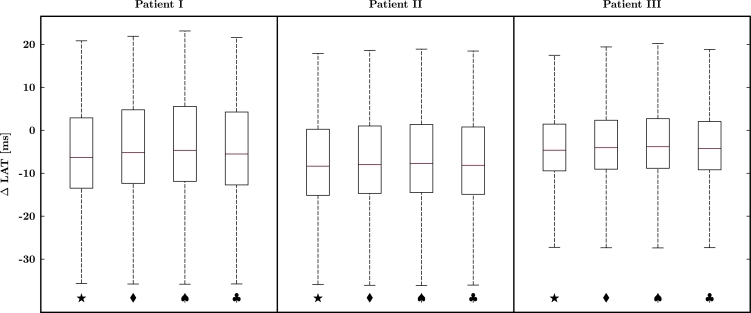
Differences in local activation times between the anisotropic electrophysiology simulations and the isotropic reference simulation within all patients; symbols ★, ♦, ♠ and ♣ indicate differences between the 2-layer, 5-layer, linear and sigmoidal interpolation simulations compared to the isotropic simulation, respectively. Boxes in the figure indicate the first and third quartile, while the red line represents the second quartile and the whiskers indicate the data range. (For interpretation of the references to colour in this figure legend, the reader is referred to the web version of this article.)

Latest activation in all patients occurred in the isotropic reference simulation with maximum LATs of 111.31, 106.43 and 88.95  ms for Patients I, II and III, respectively. When only considering the anisotropic EP simulations, maximum LATs of 102.60 and 86.11 ms were observed utilizing the linear transmural fiber interpolation function *ω*_3_(*ϕ*) in Patients I and III, respectively, while in Patient II a maximum LAT of 85.62 ms was predicted using the 2-layer transmural fiber interpolation function *ω*_1_(*ϕ*). Fig. 5 provides the individual differences in LATs between the anisotropic EP simulations using the different transmural interpolation functions and the isotropic reference simulation; symbols ★, ♦, ♠ and ♣ denote the LAT differences between the 2-layer, 5-layer, linear and sigmoidal interpolation simulations compared to the isotropic simulation, respectively. The maximum range of LAT differences was consistently observed between the anisotropic simulation using the linear interpolation function *ω*_3_(*ϕ*) and the isotropic reference simulation. These ranges were calculated as 58.98, 55.04 and 47.61 ms for Patients I, II and III, respectively. The regional activation times, an average of LATs within specific regions, were calculated at the myocardial sleeves of the PVs on the endocardium and epicardium, where only small differences were observed as a consequence of the relatively thin myocardial wall. The EP models predicted ranges of 41.11–45.98, 40.93-51.41, 42.52–58.18 and 55.26–76.07 ms for the right superior PV (RSPV), RIPV, left superior PV (LSPV) and left inferior PV (LIPV), respectively, where an increasing range corresponds to an increasing distance of the specific region from the initial stimulation site.

**Fig. 6:**
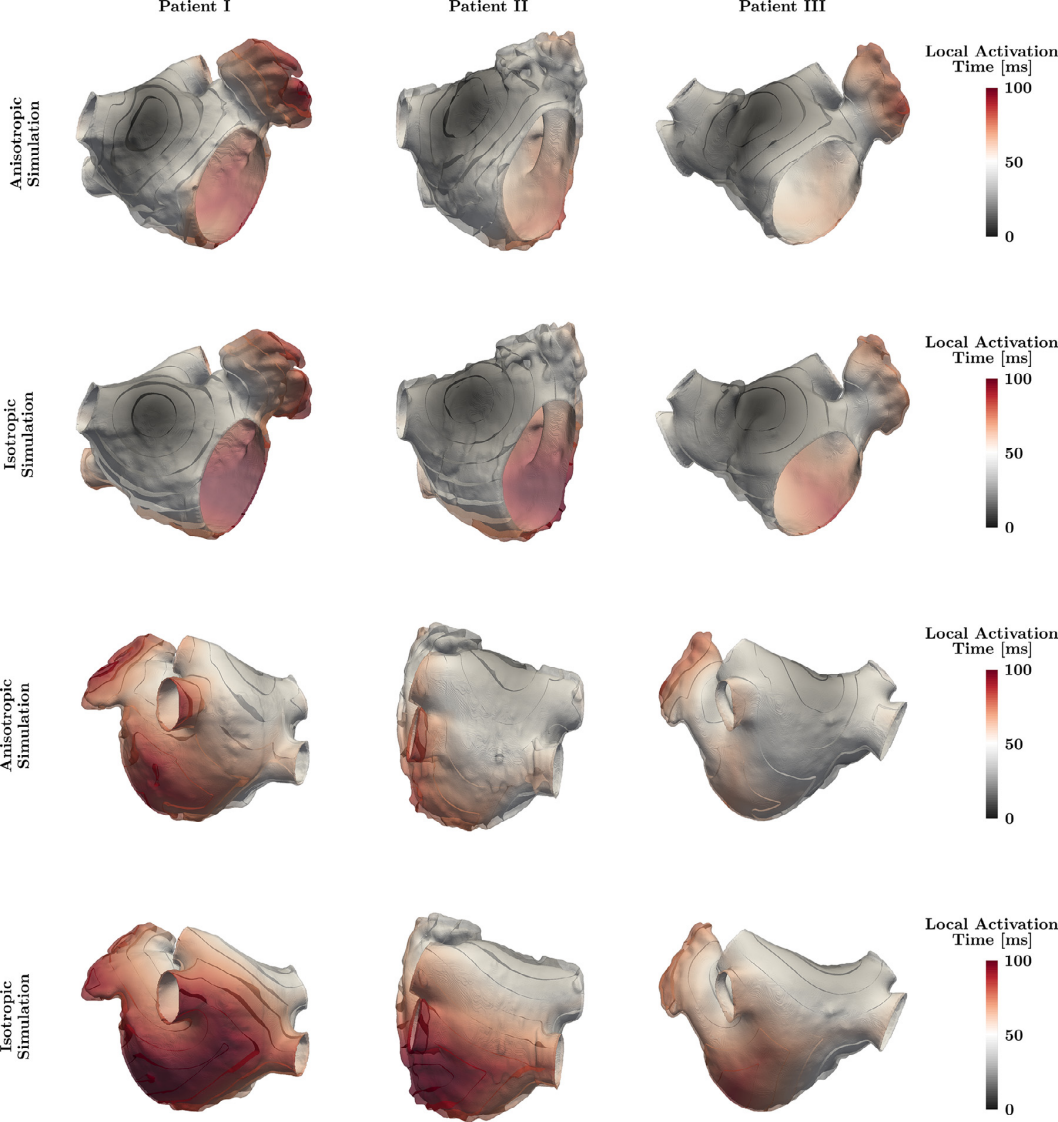
Anterior (top rows) and posterior (bottom rows) perspective of analyzed patients showing the local activation time for isotropic and anisotropic electrophysiology simulations utilizing the 2-layer transmural fiber interpolation scheme *ω*_1_(*ϕ*). Isochrones are provided in 10  ms intervals and individual patient images are not to scale.

Fig. 6 shows the anterior and posterior perspective of the anisotropic 2-layer simulation and the isotropic reference simulation for analyzed patients color-coded according to the calculated LAT. Isochrones of the LAT are provided in 10 ms intervals indicating large differences in the LAT and activation wave front shape between both EP simulations. While the isotropic reference simulation showed a circular spread of electrical activation around the stimulus region, the effect of the myocardial fiber arrangement became apparent in the anisotropic simulation leading to an elliptical activation spread. Moreover, the activation wave front exhibited a skewed shape due to different fiber directions on the endo- and epicardium.

## 4 Discussion

In this study, a novel pipeline to generate detailed personalized computational models for left atrial EP requiring a minimum degree of manual intervention was presented. A statistics-based segmentation algorithm identified the left atrial myocardium in coronary CTA images and enabled the generation of geometrical models with a varying myocardial thickness, for which a Laplace-based algorithm estimating the complex atrial fiber architecture was proposed. The modeling pipeline was applied to 3 patients and the impact of a transmurally varying fiber orientation on simulated LATs was quantified.

### 4.1 Computational model generation

The proposed statistics-based atrial segmentation algorithm extends on the method previously introduced in Bishop et al. (2016) by standardized determination of threshold values for each patient. Conventional segmentation algorithms for the atria have focused on the identification of the atrial cavity not labeling the atrial myocardium. Several studies have targeted and analyzed MRI with a relatively low image resolution (1−2 mm) when compared to the thickness of the myocardial wall in the LA (0.5−3 mm), which has made the segmentation of these images challenging (Whitaker et al., 2016), although recent progress has been made (Varela et al., 2017). As histograms in Fig. A1 indicate, the voxel intensity distributions of the ventricular myocardium are consistent across the 3 patient cases and well within the range reported for contrast-enhanced CT images (Ahn, Kim, Hur, Lee, Kim, Choe, Choi, 2009, Rodríguez-Granillo, Rosales, Degrossi, Rodriguez, 2010). Furthermore, a large intra- and interpatient variability, i.e., histograms exhibiting bimodal characteristics, in voxel intensities was observed in the blood pool reflecting a varied contrast agent concentration. This artifact results from the coronary CTA imaging acquisition, specifically from the image reconstruction, in which the voxel image was generated over the course of typically 2 consecutive heart beats changing the intensity of the contrast agent as it was pumped through the circulatory system. An identical threshold value MT was necessary in the image segmentation to obtain a continuous transition at the endocardial surface between the atrial myocardium and the contrast agent filled atrial cavity. While the lower threshold LT significantly influences the detection of viable atrial myocardium, the upper threshold UT removes structures with higher radiodensity, e.g., the vertebrae. The statistics-based image segmentation algorithm proposed herein is the first to account for the interimage variability in atrial myocardium and blood pool intensities.

Through recent progress in medical imaging and image analysis a variety of geometrical models of the LA have been generated (Seemann, Höper, Sachse, Dössel, Holden, Zhang, 2006, Jernigan, Buckner, Eischen, Cormier, 2007, Zhang, Gay, 2008, Hunter, Liu, Lu, Wang, Schilling, 2012, Krueger, Seemann, Rhode, Keller, Schilling, Arujuna, Gill, O’Neill, Razavi, Dössel, 2013, Gonzales, Sturgeon, Krishnamurthy, Hake, Jonas, Stark, Rappel, Narayan, Zhang, Segars, McCulloch, 2013, Satriano, Bellini, Vigmond, Di Martino, 2013, Adeniran, MacIver, Garratt, Ye, Hancox, Zhang, 2015, Ferrer, Sebastián, Sánchez-Quintana, Rodríguez, Godoy, Martínez, Saiz, 2015, Moyer, Norton, Ferguson, Holmes, 2015, McDowell, Zahid, Vadakkumpadan, Blauer, MacLeod, Trayanova, 2015, Zhao, Hansen, Csepe, Lim, Wang, Williams, Mohler, Janssen, Weiss, Hummel, Fedorov, 2015, Zhao, Hansen, Wang, Csepe, Sul, Tang, Yuan, Li, Bratasz, Powell, Kilic, Mohler, Janssen, Weiss, Simonetti, Hummel, Fedorov, 2017, Varela, Colman, Hancox, Aslanidi, 2016). While in the majority of computational left atrial models a completely homogeneous or regionally homogeneous myocardial wall thickness has been assumed, only more recent studies have incorporated the variation in left atrial wall thickness (Seemann, Höper, Sachse, Dössel, Holden, Zhang, 2006, Adeniran, MacIver, Garratt, Ye, Hancox, Zhang, 2015, Zhao, Hansen, Csepe, Lim, Wang, Williams, Mohler, Janssen, Weiss, Hummel, Fedorov, 2015, Zhao, Hansen, Wang, Csepe, Sul, Tang, Yuan, Li, Bratasz, Powell, Kilic, Mohler, Janssen, Weiss, Simonetti, Hummel, Fedorov, 2017, Varela, Morgan, Theron, Dillon-Murphy, Chubb, Whitaker, Henningsson, Aljabar, Schaeffter, Kolbitsch, Aslanidi, 2017). In this study the first left atrial models derived from high-resolution clinical patient data with a heterogeneous distribution of myocardial wall thickness were presented. Considering the transmural variation in myocardial fiber orientation, the left atrial wall thickness represents an important structural parameter in EP (Labarthe et al., 2013). As a consequence of the detailed representation of the left atrial myocardium, unstructured high-resolution and high-fidelity meshes of the LA were generated adopting the methods in Bishop et al. (2010) and Crozier et al. (2016). The increased FE mesh resolution reduces the numerical error in conduction velocity (Niederer et al., 2011), particularly important in fibrillation studies. The tetrahedral FE mesh volumes reported in Table 2 provide an approximation of the myocardial volume in the LA and exhibit large interpatient variation. The obtained values are consistent with the volumes of the original image segmentations showing errors smaller than 1.71%. Zhao et al. (2017) computed the left atrial tissue volume of a human female cadaver as 30.70 cm^3^ utilizing *ex vivo* contrast-enhanced MRI. The differences to measurements in Table 2 could potentially be explained by the differences in left atrial wall thickness observed when measured using different imaging modalities (Whitaker et al., 2016).

### 4.2 Fiber generation algorithm

The major advantage of the proposed method to generate detailed personalized fiber architectures in computational models of the LA over existing approaches is the high degree of automation. Although rule-based and atlas-based approaches presented in the literature reflect the underlying myocardial fiber orientation (Werner, Sachse, Dössel, 2000, Hermosillo, 2008, Krueger, Schmidt, Tobón, Weber, Lorenz, Keller, Barschdorf, Burdumy, Neher, Plank, Rhode, Seemann, Sanchez-Quintana, Saiz, Razavi, Dössel, 2011, Labarthe, Coudiere, Henry, Cochet, 2012, Satriano, Bellini, Vigmond, Di Martino, 2013, McDowell, Vadakkumpadan, Blake, Blauer, Plank, MacLeod, Trayanova, 2012), they suffer from either increased intra- and interobserver variability as well as limited reproducibility or are not suited for large patient cohorts. The novel method presented herein combines the advantages of both approaches using an automated transfer of predefined landmarks from an average atrial geometry to a personalized atrial geometry and *a priori* definition of certain rules to generate fibers. This combination of individual ideas results in a standardized procedure providing more consistent estimates of the left atrial fiber architecture. Moreover, the novel approach facilitates the consistent development of both surface and volume fibers for which specific transmural interpolation functions can be applied.

The visual comparison between the estimated left atrial fiber architecture and the morphological images in Fig. 4 illustrates a qualitative structural correspondence for all major fiber bundles. Although difficult, qualitative similarities in key regions are observed when the estimated *in vivo* left atrial fiber architectures obtained using the proposed algorithm are visually compared to fiber architectures reported in the literature obtained using *ex vivo* imaging modalities. The estimated left atrial myofiber orientations qualitatively match the calculated left atrial myofiber orientations in the posterior regions reported in Zhao et al. (2015) and Zhao et al. (2017) derived from a single specimen using micro-CT and contrast-enhanced MRI, respectively. Pashakhanloo et al. (2016) characterized the left atrial myofiber architecture using diffusion tensor MRI in 8 specimens revealing consistent topologies for the major bundle structures with the estimated left atrial myofiber architecture. Moreover, Pashakhanloo et al. (2016) reported on the detailed transmural variation of fiber angles in 2 specimens utilizing 4 different regions of interest concluding either a 2-layer myofiber structure or no transmural myofiber variation.

### 4.3 Electrophysiology simulations

The performed EP simulations predicted consistently longer maximum LATs for the isotropic reference simulations when compared to anisotropic simulations. The alignment of the fiber orientation with the activation direction leads to faster anisotropic simulations, consistent with findings in Krueger et al. (2011) and Krueger et al. (2013) comparing a homogeneous isotropic and a heterogeneous anisotropic biatrial model. Similar LATs for anisotropic simulations using different transmural fiber interpolation functions within each patient suggest a negligible effect of the transmural fiber architecture on the activation pattern. The obtained maximum LATs in all patients for physiological conditions, i.e., anisotropic EP simulations, were slightly longer than clinically measured LATs in the LA for patients with an AF history (cf., e.g., 86 ± 19, 81 ± 10 and 75.4 ± 16.6 ms in Lemery et al. (2007), Chang et al. (2007) and Choi et al. (2010), respectively). This could potentially be explained by either the insufficient resolution in clinical measurements, particularly in the LAA, or the assumptions of using a single stimulus site as well as homogeneous cellular and tissue properties in EP simulations. Moreover, AF-induced electrical and structural remodeling can prolong the maximum LAT in the LA (Schotten et al., 2011). The calculated regional activation time ranges correspond with the measured ranges in Lemery et al. (2007) (cf., 44, 47–52, 58–68 and 70–80 ms for the RSPV, RIPV, LSPV and LIPV, respectively, following the subtraction of the LAT at earliest LA activation) providing an additional EP model validation. The isotropic EP simulations were conducted utilizing anisotropic monodomain conductivity tensors combined with random fiber architectures to remove the preferential conduction on the macroscopic scale.

### 4.4 Limitations

The proposed framework was designed to automatically generate personalized computational EP models of the LA from clinical and morphological data, while full personalization applies to the geometry only. There are inherent assumptions and limitations associated with such a challenging task discussed below.

The generation of geometrical models from coronary CTA images via statistics-based image segmentation relies on 3 assumptions. First, atrial and ventricular myocardium exhibit comparable radiodensities, i.e., voxel intensities in the myocardium of the LA and the left ventricle are similar. Second, structures in the proximity of the LA have different voxel intensities such that an epicardial boundary of the LA can be detected. Third, a sufficiently thick myocardial wall in the LA assumed to be minimum 1 voxel so that partial volume effects and motion artifacts have a negligible impact on the estimate of the myocardium. Despite these assumptions, estimated anatomical wall thicknesses in our models are comparable with manual measurements in CT images (Whitaker et al., 2016). Improvements in current CT technology, in particular the increased use of dual energy source scanners, will result in increased image resolution and soft tissue contrast. This will further minimize the impact of errors introduced by these assumptions.

The complex left atrial fiber architecture was qualitatively estimated based on morphological images as opposed to measured from micro-CT (Varela, Zhao, Aslanidi, 2013, Zhao, Hansen, Csepe, Lim, Wang, Williams, Mohler, Janssen, Weiss, Hummel, Fedorov, 2015, Stephenson, Atkinson, Kottas, Perde, Jafarzadeh, Bateman, Iaizzo, Zhao, Zhang, Anderson, Jarvis, Dobrzynski, 2017), diffusion tensor MRI (Pashakhanloo et al., 2016) or contrast-enhanced MRI (Zhao et al., 2017). Distinct challenges arise when attempting to assess the atrial fiber structure using diffusion tensor MRI due to cardiac motion and a thin-walled atrial phenotype, hence this has only been performed *ex vivo*, where an interpatient analysis has demonstrated that the main features of the fiber orientation are preserved (Pashakhanloo et al., 2016). The proposed algorithm was developed to represent all prominent fiber structures, but some degree of simplification was required. The fiber architecture in the septal portion of the LA, in particular the right atrial portion, is highly complex and subjected to large interpatient variability making it impossible to define a standard myofiber orientation. Therefore, a continuation of the fiber direction present in the anterior right atrial septum was chosen as a standardization strategy. In goat models the presence of persistent AF altered the myofiber architecture of the LA (Maesen et al., 2013), while in a limited number of human atria no significant change in the myofiber architecture was observed between AF patients and patients with no specific atrial pathology (Pashakhanloo et al., 2016). Further measurements of the fiber orientation in AF patients are required to confirm and quantify potential changes associated with AF.

Homogeneous EP at the cellular and tissue level was assumed throughout the LA providing standardized FE simulation conditions isolating the effect of the transmural fiber architecture. Different regions in the LA such as the BB, PVs, LAA and MV exhibit distinct electrophysiological properties and would need to be included to obtain a more physiological activation pattern (Dössel et al., 2012). In addition, a single electrical stimulus was applied in the area of the BB, while the electrical signal might enter the LA on multiple sites (Markides et al., 2003). The novel pipeline focused on the LA, arguably the simpler atrium in terms of anatomy and fiber architecture, however, its critical role in AF made it the focus of attention.

## 5 Conclusion

The presented modeling pipeline to generate detailed personalized computational models for left atrial EP requires a minimum degree of manual intervention facilitating the consistent development of both surface and volumetric FE models including the heterogeneous myocardial wall thickness. The complex left atrial fiber architecture within the 3-dimensional FE models derived from clinical data was estimated using a novel algorithm and qualitatively represents the most dominant fiber structures visible in morphological studies. The influence of the transmural fiber orientation on the LAT was investigated utilizing EP simulations and only minor differences were observed suggesting a negligible effect.
